# Constitutive systemic inflammation in Shwachman-Diamond Syndrome

**DOI:** 10.1186/s10020-025-01133-5

**Published:** 2025-02-28

**Authors:** Giuseppe Sabbioni, Elisabetta D’Aversa, Giulia Breveglieri, Maria Teresa Altieri, Christian Boni, Anna Pegoraro, Alessia Finotti, Roberto Gambari, Giovanna D’Amico, Antonio Vella, Giuseppe Lippi, Marco Cipolli, Valentino Bezzerri, Monica Borgatti

**Affiliations:** 1 Department of Life Sciences and Biotechnology, University of Ferrara, Ferrara, Italy; 2 Department of Translational Medicine, University of Ferrara, Ferrara, Italy; 3 Cystic Fibrosis Center, Azienda Ospedaliera Universitaria Integrata, Verona, Italy; 4 Centro Tettamanti, Fondazione IRCCS San Gerardo dei Tintori, Monza, Italy; 5 Unit of Immunology, Azienda Ospedaliera Universitaria Integrata, Verona, Italy; 6 Section of Clinical Biochemistry, Department of Engineering for Innovation Medicine, University of Verona, Verona, Italy; 7 Department of Life Sciences, Health, and Health Care Professions, Link Campus University, Rome, Italy; 8 Interuniversity Consortium for Biotechnology (CIB), Trieste, Italy

**Keywords:** Shwachman-Diamond Syndrome, Acute myeloid leukemia, mTOR, Inflammation

## Abstract

**Background and purpose:**

Shwachman-Diamond Syndrome (SDS) is an autosomal recessive disease belonging to the inherited bone marrow failure syndromes and characterized by hypocellular bone marrow, exocrine pancreatic insufficiency, and skeletal abnormalities. SDS is associated with increased risk of developing myelodysplastic syndrome (MDS) and/or acute myeloid leukemia (AML). Although SDS is not primarily considered an inflammatory disorder, some of the associated conditions (e.g., neutropenia, pancreatitis and bone marrow dysfunction) may involve inflammation or immune system dysfunctions. We have already demonstrated that signal transducer and activator of transcription (STAT)-3 and mammalian target of rapamycin (mTOR) were hyperactivated and associated with elevated IL-6 levels in SDS leukocytes. In this study, we analyzed the level of phosphoproteins involved in STAT3 and mTOR pathways in SDS lymphoblastoid cells (LCLs) and the secretomic profile of soluble pro-inflammatory mediators in SDS plasma and LCLs in order to investigate the systemic inflammation in these patients and relative pathways.

**Methods:**

Twenty-six SDS patients and seven healthy donors of comparable age were recruited during the programmed follow-up visits for clinical evaluation at the Verona Cystic Fibrosis Center Human. The obtained samples (plasma and/or LCLs) were analyzed for: phosphoproteins, cytokines, chemokines and growth factors levels by Bio-plex technology; microRNAs profiling by next generation sequencing (NGS) and microRNAs expression validation by Real Time-PCR (RT-PCR) and droplet digital PCR (ddPCR) .

**Results:**

We demonstrated dysregulation of ERK1/2 and AKT phosphoproteins in SDS, as their involvement in the hyperactivation of the STAT3 and mTOR pathways confirmed the interplay of these pathways in SDS pathophysiology. However, both these signaling pathways are strongly influenced by the inflammatory environment. Here, we reported that SDS is characterized by elevated plasma levels of several soluble proinflammatory mediators. In vitro experiments show that these pro-inflammatory genes are closely correlated with STAT3/mTOR pathway activation. In addition, we found that miR-181a-3p is down-regulated in SDS. Since this miRNA acts as a regulator of several pro-inflammatory pathways such as STAT3 and ERK1/2, its down-regulation may be a driver of the constitutive inflammation observed in SDS patients.

**Conclusions:**

The results obtained in this study shed light on the complex pathogenetic mechanism underlying bone marrow failure and leukemogenesis in SDS, suggesting the need for anti-inflammatory therapies for SDS patients.

**Supplementary Information:**

The online version contains supplementary material available at 10.1186/s10020-025-01133-5.

## Introduction

Shwachman-Diamond Syndrome (SDS) (OMIM #260,400) is a rare inherited bone marrow failure syndrome (IBMFS), with an incidence of about 1:75,000–1:168,000 (Han et al. 2023). More than 90% of patients carry mutations in the Shwachman-Bodian-Diamond Syndrome (*SBDS*) gene on chromosome 7q11, though mutations in the *DNAJC21, EFL1*, and *SRP54* genes have been associated with SDS-like conditions (Kawashima et al. 2023a). SDS affects multiple organs and is characterized by exocrine pancreatic insufficiency, skeletal abnormalities and bone marrow failure, which is in turn associated with development of myelodysplastic syndrome (MDS) and acute myeloid leukemia (AML) (Han et al. 2023). In general, SDS patients already show mild to severe neutropenia in the early stages of life, whereas anemia and thrombocytopenia are less frequent. In SDS, bone marrow failure results in severe impairment of myeloid lineage, with decreased CD34^+^ cell count and impaired maturation of myeloid progenitors at the myelocyte and metamyelocyte stages (Mercuri et al. 2015). In addition, lymphoid lineage impairment has also been reported in SDS, with lower numbers of circulating B cells, T-lymphocytes and natural killer cells (Dror et al. 2001). Since no therapy aimed at reducing the emergence of MDS/AML has so far been developed for SDS and other IBMFS, allogeneic hematopoietic stem cell transplant remains the sole option in these cases (Bezzerri and Cipolli 2019).

Inflammation has been poorly investigated in SDS to date. Furutani et al. (Furutani et al. 2020) described 5 patients with SDS showing different inflammatory conditions, such as blepharoconjunctivitis, arthritis, chronic recurrent multifocal osteomyelitis (CRMO) and scleroderma, not previously reported in these subjects; the authors hypothesized that such manifestations might be part of SDS phenotypic symptoms. Interestingly, elevated levels of pro-inflammatory cytokines and chemokines correlated with the NF-κB pathway, such as chemokine (C–C motif) ligand (CCL)16, CCL21 and interleukin (IL)−8, were found in these patients. Immunohistochemical analyses on an English cohort of 15 patients with SDS identified duodenal inflammatory features in most cases, with increased numbers of lymphocytes, CD20^+^ B cells, macrophages and eosinophils, mostly localized in the deep lamina propria, around crypt bases (Shah et al. 2010). Targeted depletion of *SBDS* in murine pancreas resulted in p53 increase, suggesting early activation of cellular senescence. Consistent with this hypothesis, senescent acinar cells showed hyperactivation of NF-kB transcription factor and elevated release of TGF-β (Tourlakis et al. 2015). In addition, we have already demonstrated that signal transducer and activator of transcription (STAT)−3 and mammalian target of rapamycin (mTOR) were hyperactivated, this being associated with elevated IL-6 levels in SDS leukocytes (Bezzerri et al. 2016; Vella et al. 2020). Importantly, the up-regulation of STAT3 and mTOR plays a key role in AML development, neutrophil differentiation and osteogenesis (Vella et al. 2020; O'Shea et al. 2013). In addition, STAT3 also regulates the transcription of inflammatory markers, including IL-6, which in turn may activate STAT3 pathways, generating a positive, self-perpetuating loop, often found to be overactivated in AML (Vella et al. 2020).

MicroRNAs (miRNAs) are small non-coding, regulatory RNAs able to bind to 3’-untranslated regions (UTR) of messenger RNA (mRNA). They regulate gene expression by forming the RNA-induced silencing complex (RISC), which may promote either mRNA degradation or translational disruption (Swarts et al. 2014). It has been widely demonstrated that one miRNA can regulate many genes. Of note, dysregulated miRNA expression has already been linked to leukemogenesis (Stavast et al. 2018). In addition, miRNAs can regulate a variety of cellular processes such as cell proliferation and apoptosis. Most importantly, miR-181a has recently been described as a potent regulator of STAT3 in T-cell large granular lymphocyte leukemia (Assmann et al. 2022). In particular, miR-181a-3p expression has been proposed as a diagnostic marker for AML, up-regulation of miR-181a-3p expression being correlated with favorable prognosis. Additionally, up-regulated miR-181a-3p can inhibit NF-κB activation (Qiang et al. 2020), reducing cell proliferation rate in cancer. The inhibitory activity of miR-181a-3p on NF-kB signaling has also been observed in endothelial cells, retarding atherosclerosis progression (Su et al. 2019).

In this study, we analyzed the level of phosphoproteins involved in STAT3 and mTOR pathways in lymphoblastoid cells (LCLs), obtained from healthy donors and SDS patients. We found that the phosphorylation of ERK1/2 and AKT is dysregulated in SDS. Since STAT3 and mTOR signaling pathways are strongly influenced by the inflammatory environment, the secretomic profile of soluble pro-inflammatory mediators was analyzed in SDS patients. Results indicated higher expression of several markers of inflammation in SDS, compared with age-matched healthy donors. In parallel, we observed decreased expression of the pro-inflammatory miRNA miR-181a-3p, which may explain the dysregulation of the mTOR/STAT3 pathways in SDS. Taken together, our results shed light on the complex molecular mechanisms underlying the pathogenesis of SDS, suggesting the need for the development of tailored anti-inflammatory therapies for patients with SDS.

## Methods

### Human subjects

Human samples were obtained and analyzed in compliance with the Declaration of Helsinki, after written consent. All protocols were approved by the Ethics Committee of Azienda Ospedaliera Universitaria Integrata (Verona, Italy: approval No. 4182 CESC). Twenty-six SDS patients and seven healthy donors of comparable age were recruited during the programmed follow-up visits for clinical evaluation at the Verona Cystic Fibrosis Center. SDS patients (in the absence of treatment with G-CSF, other bone marrow stimulating factors and stem cell transplant) were included only if they carried biallelic *SBDS* mutations, without MDS/AML, as indicated in Table 1, where genotype, ANC (cells/mm^3^) and phenotype were reported for each patients. No patients were experiencing infections at the enrollment. During the outpatient visit scheduled at the local phlebotomy center, one additional blood sample (5 ml) was obtained.

**Table 1:** Clinical data of recruited SDS patients

UPN	SEX	AGE	Genotype	ANC (cells/mm^3^)	Phenotype	MDS/AML
1	M	27	258 + 2 T > C / 183–184 TA > CT	800	PI, thrombocytopenia, bone malformations	No
6	M	M	258 + 2 T > C / 101 A > T	2940	PI, thrombocytopenia, bone malformations	No
13	M	19	258 + 2 T > C / 183–184 TA > CT + 258 + 2 T > C	1480	PI, thrombocytopenia, bone malformations	No
26	M	16	258 + 2 T > C / 183–184 TA > CT	490	PI, thrombocytopenia, bone malformations	No
37	F	10	258 + 2 T > C / 183–184 TA > CT	250	PI, bone malformations	No
47	M	12	258 + 2 T > C / 183–184 TA > CT	600	PI, thrombocytopenia, bone malformations	No
52	M	10	258 + 2 T > C / 183–184 TA > CT + 258 + 2 T > C	1080	PI, bone malformations	No
56	F	15	258 + 2 T > C / 183–184 TA > CT	1340	PI, bone malformations	No
57	F	40	258 + 2 T > C / 63 G > C	470	PI, thrombocytopenia, bone malformations	No
58	M	12	258 + 2 T > C / 183–184 TA > CT	1390	PI, thrombocytopenia, bone malformations	No
63	M	15	258 + 2 T > C / 183–184 TA > CT + 258 + 2 T > C	1290	PI, bone malformations	No
65	M	19	258 + 2 T > C / 183–184 TA > CT	1240	PI, thrombocytopenia, bone malformations	No
66	M	23	258 + 2 T > C / 183–184 TA > CT	2400	PI, thrombocytopenia, bone malformations	No
68	M	N.A	258 + 2 T > C / 183–184 TA > CT	1907	PI, thrombocytopenia, bone malformations	No
69	F	8	258 + 2 T > C / 183–184 TA > CT	1170	PI, thrombocytopenia, bone malformations	No
72	M	28	258 + 2 T > C / 183–184 TA > CT	430	PI, thrombocytopenia, bone malformations	No
73	F	7	258 + 2 T > C / 183–184 TA > CT	2160	PI, thrombocytopenia, bone malformations	No
74	M	9	258 + 2 T > C / 183–184 TA > CT	1320	PI, thrombocytopenia, bone malformations	No
75	F	N.A	258 + 2 T > C / 183–184 TA > CT	920	PI, thrombocytopenia, bone malformations	No
80	M	7	258 + 2 T > C / 183–184 TA > CT	790	PI, thrombocytopenia, bone malformations	No
87	M	18	258 + 2 T > C / 183–184 TA > CT	1290	PI, thrombocytopenia, bone malformations	No
89	M	N.A	258 + 2 T > C / 258 + 533–459 + 403del	800	PI	No
94	F	19	258 + 2 T > C / 183–184 TA > CT	1750	PI, bone malformations	No
104	M	10	258 + 2 T > C / 183–184 TA > CT	300	PI, thrombocytopenia, bone malformations	No
106	M	36	258 + 2 T > C / 183–184 TA > CT	1200	PI, bone malformations	No
108	M	17	258 + 2 T > C / 183–184 TA > CT	1438	bone malformations	No

UPN, unique patient number; ANC, absolute neutrophil count; PI, pancreatic insufficiency

### LCLs cultures

Lymphoblastoid cell lines (LCLs) from SDS patients and healthy donors were obtained as previously described (Roncella et al. 1990). Briefly, B cells were isolated from peripheral blood by use of the RosetteSep B Lymphocyte Kit (Miltenyi Biotech, Bergisch Gladbach, Germany). B cells were characterized by flow cytometry, evaluating the expression of the common leukocyte antigen CD45 and the pan-B lymphocyte antigen CD19. B cells (3 × 10^6^) were infected with Epstein Barr virus (EBV) derived from marmoset blood leukocyte B95.8 virus-producer cell lines for 18 h, in 3 ml RPMI-1640 (Sigma-Aldrich, St Louis, MO), supplemented with 10% FBS. Once generated, LCLs with a comparable number of cell culture passages were incubated for 24 h in RPMI-1640 medium (Sigma-Aldrich, St. Louis, MO, USA), supplemented with 10% fetal bovine serum (FBS) (Sigma, St. Louis, MO, USA). In order to inhibit mTOR phosphorylation, the clinically approved rapamycin derivative everolimus (RAD001) was employed. Briefly, SDS LCLs were incubated in the absence (DMSO as control) or in the presence of 350 nM everolimus for 24 h. Then, supernatants were collected and analyzed using the Bio-Plex Human Cytokine 27-plex Assay (Bio-Rad, Hercules, CA).

### Phospho flow analysis

LCLs obtained from healthy donors and SDS patients were seeded at 2.5 × 10^5^ cells in 24-well plates, in complete RPMI-1640 medium, and incubated at 37° C for 24 h. Cells were washed with PBS, then fixed and permeabilized using the eBioscience Intracellular Fixation & Permeabilization Buffer Set (ThermoFisher, Waltham, MA), following the manufacturer’s instructions. Subsequently, cells were washed twice in flow buffer (PBS, pH 7.2, with 0.2% BSA and 0.09% sodium azide) and then stained with anti-pS727-STAT3-PE, anti-Y705-STAT3-PE, anti-p-S2448-mTOR-PE or isotype control-PE for 30 min (all antibodies supplied by Becton–Dickinson Biosciences, Franklin Lakes, NJ). Cells were washed and acquired on a 13-color DxFLEX flow cytometer (Beckman Coulter, Indianapolis, IN). Doublets were discriminated by plotting FSC height vs FCS area, before isolating cells on the basis of their forward and side scatter properties (an example of gating strategy was reported in Additional file 1: Figure S1). All acquired data files were analyzed using Kaluza software, version 2.2 (Beckman Coulter, Indianapolis, IN). Results were reported as fold-change in median fluorescence intensity relative to healthy controls.

### Phosphoprotein analysis

Phosphoproteins were analyzed by Luminex^®^ xMap^®^  technology (Bio-Rad, Hercules, CA), based on multiplex sandwich bead immunoassays. LCLs extracts were analyzed   by phosphoprotein assay (Bio-Rad, Hercules, CA) according to the manufacturer's recommendations. Briefly, protein extracts were transferred into 96-well dishes and diluted with 25 μl buffered solution. Fluorescent capturing beads coupled to antibodies were mixed, added to each well, and incubated overnight. Following incubation, the plates were washed and incubated with biotinylated antibodies, fixing each target protein. Streptavidin–phycoerythrin solution was then added. The analysis comprised double laser fluorescence detection, allowing simultaneous identification of the target protein through the red fluorescence emission signal of the bead and quantification of the target protein through fluorescence intensity of phycoerythrin. Results were recorded as mean fluorescence intensities and compared to negative controls.

### Analysis of cytokines, chemokines and growth factors

Proteins released into plasma or cell culture supernatants were measured using the Human Cytokine 27-plex Assay (Bio-Rad, Hercules, CA), as indicated by the manufacturer. The assay allows the multiplexed quantitative measurement of 27 cytokines/chemokines (FGF basic, Eotaxin, G-CSF, GM-CSF, IFN-γ, IL-1β, IL-1ra, IL-2, IL-4, IL-5, IL-6, IL-7, IL-8, IL-9, IL-10, IL-12 (p70), IL-13, IL-15, IL-17A, IP-10, MCP-1 (MCAF), MIP-1α, MIP-1β, PDGF-BB, RANTES, TNF-α, VEGF), in a single well. A 50 μl specimen of cytokine standards or samples was incubated with 50 μl of anti-cytokine conjugated beads in a 96-well plate for 30 min, at room temperature with shaking. The plate was washed for three times with 100 μl of Wash Buffer; 25 μl of diluted detection antibody were added to each well, and the plate was incubated for 30 min at room temperature with shaking. After three washes, 50 μl of streptavidin–phycoerythrin were added, and the plate was incubated for 10 min at room temperature with shaking. Finally, the plate was washed for three times, beads were suspended in Assay Buffer, and the plate was examined by Luminex^®^ xMap^®^ technology. Data were collected and analyzed by the Bio-Plex Manager Software (Bio-Rad, Hercules, CA) (Guerrini et al. 2014).

### Small RNA next generation sequencing (NGS)

NGS experiments were performed at the Laboratory for Technologies of Advanced Therapies (LTTA), Ferrara University. Small RNA libraries were prepared using TruSeq^®^ Small RNA Library PrepKit v2 (Illumina, RS-200-0012/24/36/48), according to the manufacturer’s indications. Briefly, 35 ng of purified RNA were linked to RNA 3’ and 5’ adapters, converted to cDNA and amplified by means of Illumina primers containing unique indexes for each sample. Libraries were quantified using the Agilent Bioanalyzer and High Sensitivity DNA Kit (Agilent, 5067–4626); equal amounts of libraries were then pooled together and submitted to size selection, keeping only fragments between 130 and 160 bp. After ethanol precipitation, the library pool was quantified by means of the Agilent Bioanalyzer and High Sensitivity DNA Kit, denatured, diluted to 1.8 pM and sequenced, using the Illumina NextSeq500 platform and NextSeq^®^ 500/550 High Output Kit v2 (75 cycles) (Illumina, FC-404–2005). Raw base-call data generated from the Illumina NextSeq 500 system were demultiplexed automatically by the Illumina BaseSpace Sequence Hub (https://basespace.illumina.com/home/index) and converted to FASTQ format. After a quality check using the FastQC tool (https://www.bioinformatics.babraham.ac.uk/projects/fastqc/), adapter sequences were trimmed by Cutadapt (http://cutadapt.readthedocs.io/en/stable/index.html). During this step, sequences shorter than 10 nucleotides were removed. Read mapping was done by the STAR algorithm (https://www.ncbi.nlm.nih.gov/pubmed/23104886), the reference genome being composed of human microRNA sequences from miRbase 22 (http://www.mirbase.org/). A raw mapped read count was carried out with the HTSeq-count script from HTSeq tools (http://www-huber.embl.de/HTSeq/doc/overview.html); raw counts were normalized using the DESeq2 bioconductor package (http://bioconductor.org/packages/release/bioc/html/DESeq2.html).

### miRNA extraction and droplet digital RT-PCR quantification

Healthy and SDS LCLs were collected by centrifugation at 1500 rpm for 10 min, at 4 °C. The cellular pellets were washed twice with PBS and lysed with TRI reagent (Sigma Aldrich, St. Louis, Missouri). RNA extraction was conducted following the manufacturer's instructions, and the isolated RNA was washed once with cold 75% ethanol. After washing, it was dried and dissolved in nuclease-free pure water; 300 ng of total RNA were reverse transcribed using the TaqMan™ miRNA Reverse Transcription Kit (Thermo Fisher Scientific, MA) in a final volume of 30 µL, according to the manufacturer's instructions. The cDNA thus obtained was stored at − 80 °C until the time of use. For miRNA droplet digital PCR (ddPCR), the reaction mix was prepared by addition of Supermix for Probes (no dUTP) 2X (Bio-Rad, Hercules, CA), 20X TaqMan™ miRNA assay (Thermo Fisher Scientific, MA) and cDNA. Droplets were automatically generated using an Automated Droplet Generator (AutoDG) (Bio-Rad, Hercules, CA), the emulsion thus generated being amplified by the following temperature control procedure: 95 °C for 10 min, 95 °C for 15 s, and 60 °C for 1 min. Steps 2 and 3 were repeated for 40 cycles, at a ramp rate of 2.5 °C/s. A final step at 98 °C for 10 min was added, to cut off polymerase enzyme activity. Amplified droplets were analyzed for their fluorescence content, on the QX200 Droplet Digital PCR system. Data analysis was performed using QuantaSoft software, version 1.7.4 (Bio-Rad, Hercules, CA).

### Functional enrichment analysis

Functional enrichment analysis was performed by ShinyGO 0.77 (SDSU), the input being the IDs of genes coding for the inflammatory proteins found at higher concentrations in peripheral blood plasma of SDS patients. Results were sorted by false discovery rate (FDR), in ascending order. The Kyoto Encyclopedia of Genes and Genomes (KEGG) was chosen as the pathway database for the analysis, setting the FDR cutoff at 0.05.

### Statistical analysis

The Shapiro–Wilk test of normality was used to assess distribution of our samples. Once normality of distribution was confirmed, parametric tests were employed to analyze different groups. An unpaired Student’s t-test was used for comparisons between control (Ctrl) and SDS-derived samples. Comparisons between different tissues obtained from the same donor, or between cells incubated with *vs* without everolimus, were made by paired Student’s t-test. A *p* value of < 0.05 was considered as statistically significant. All statistical analyses were conducted using GraphPad Prism for Windows, version 6.07 (GraphPad Software, CA).

## Results

### Phosphoproteins involved in the STAT3 and mTOR signaling pathways in SDS

mTOR is a serine/threonine kinase that contributes to regulating a plethora of cellular processes such as protein synthesis, growth, proliferation, metabolism, aging, regeneration, and autophagy (Liu and Sabatini 2020). STAT3 has been reported as a direct substrate for mTOR, which can promote STAT3 phosphorylation both at residue tyrosine 705 and at serine 727, showing a crosstalk between these signaling pathways (Vella et al. 2020; Yokogami et al. 2000; Dodd et al. 2015; Jhaveri et al. 2016). These existing data suggest the need to explore a possible role of phosphoproteins activated downstream of these signaling pathways in SDS patients.

**Fig. 1:**
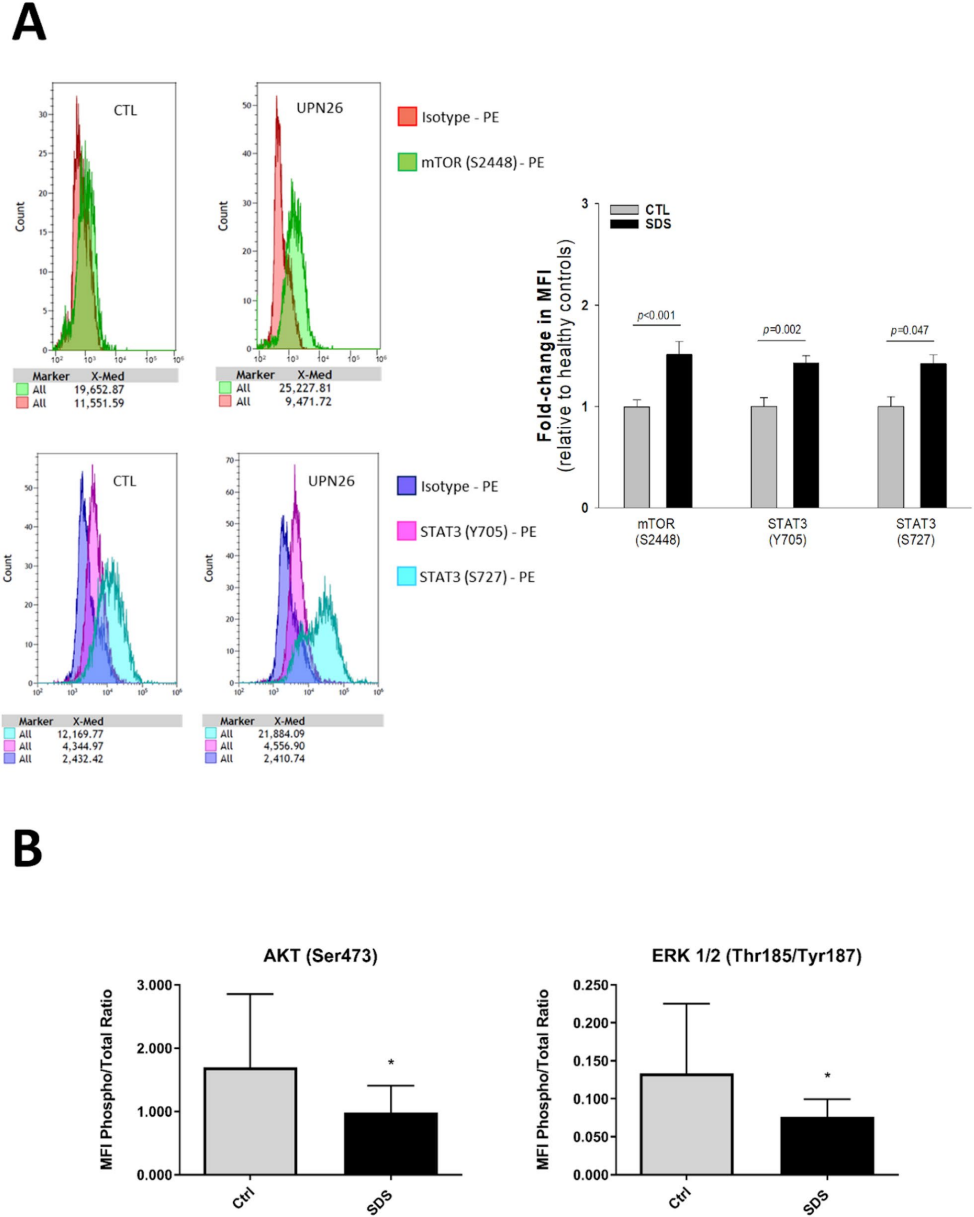
Analysis of phosphoproteins in LCLs obtained from SDS and healthy donors. LCLs from SDS patients or from healthy control subjects were cultured with the same culture length and passages in RPMI-1640 medium supplemented with 10% FBS. Successively, cells protein extracts were used to perform Phospho-Flow (**A**) and Luminex^®^ xMap^®^ (**B**) assays. (**A**): After fixation and permeabilization, cells were stained with specific PE-conjugated antibody against mTOR (S2448) or STAT3 (Y705 or S727). Flow cytometry analysis was then performed. Isotype PE-conjugated antibody was employed as negative control. The graphs on the left are representative of a Phospho-Flow analysis performed on LCLs obtained from a SDS patient (UPN26) compared with a healthy donor (CTL). The histogram on the right shows the fold-change, relative to healthy controls, of the normalized median fluorescence intensity (MFI) of mTOR (S2448, n = 9) and STAT3 (Y705 and S727, n = 6 each) in LCL from SDS patients (UPN26, UPN37, UPN75, black bars) and healthy donors (light grey bars). (**B**): The histograms show, for each analyte, the MFI ± SEM values of the phosphorylated protein on the total (MFI Phospho/Total Ratio) detected in healthy donors (light grey bars, n = 6) and SDS (black bars, n = 10) derived samples. The phosphoproteins analyzed are AKT and ERK1/2. The phosphorylated residue detected is indicated in parentheses next to each protein. * = p < 0.05; ** = p < 0.01; *** = p < 0.001

As shown in Fig. 1A, mTOR (S2448) and STAT3 (Y705 and S727) were hyperphosphorylated in LCLs obtained from SDS patients, in comparison with healthy donors. On the contrary, phosphorylated AKT (S473) and ERK 1/2 (T185/Y187) levels were significantly reduced in SDS cells (Fig. 1B). The overactivation of the mTOR pathway (Fig. 1A) could potentially be responsible for reduced levels of ERK1/2 phosphorylation (Fig. 1B) in SDS, through an already established cross-inhibition: since mTOR signaling was already found to be up-regulated in SDS (Bezzerri et al. 2016; Vella et al. 2020), ERK1/2 inhibition could act as a compensatory process, balancing some cellular programs such as survival and proliferation (Mendoza et al. 2011; Aksamitiene et al. 2012; Gkouveris et al. 2014). Additionally, reduced phospho-AKT levels may justify the increased phosphorylation of STAT3 in Tyr705 and Ser727 residues (Bian et al. 2018).

### Secretomic profile in SDS plasma and LCLs

SDS patients are more vulnerable to infections and inflammatory processes compared with healthy individuals (Furutani et al. 2020). Furthermore, the mTOR and STAT3 pathways are hyperactivated in SDS (Bezzerri and Cipolli 2019; Bezzerri et al. 2016). The activation of these two pathways can be strongly influenced by the inflammatory environment. For instance, STAT3 pathway hyperactivation leads to the transcription of IL-6, which in turn binds to its receptor and thus further activates the STAT3 signaling pathway, triggering a self-sustaining feedback loop (Wake and Watson 2015; Wang and Sun 2014; Conciatori et al. 2018; Banerjee et al. 2019).

**Fig. 2:**
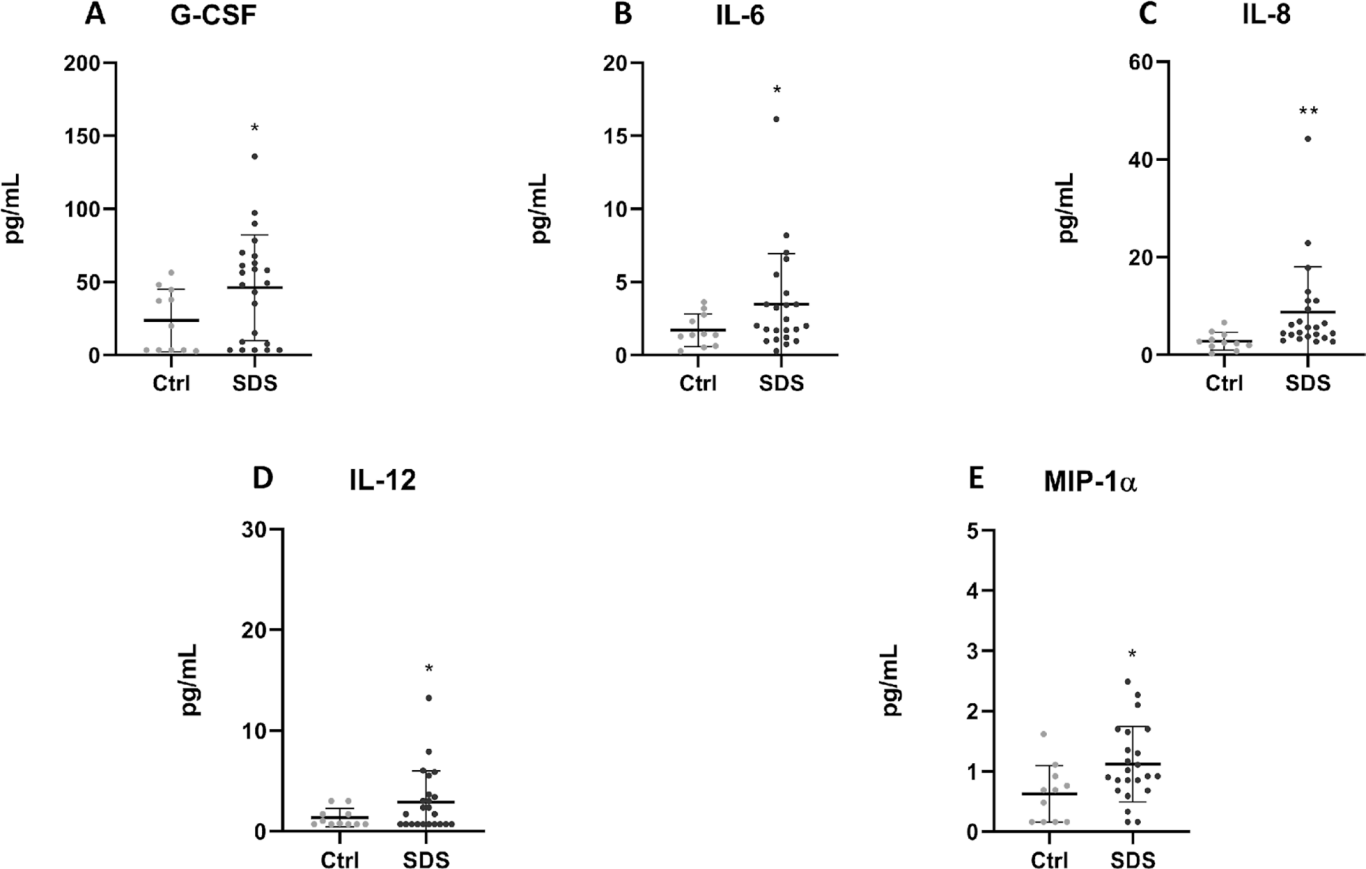
Expression profile of cytokines and chemokines in plasmas obtained from peripheral blood of healthy subjects and SDS patients. The scatter plots show the concentration, in pg/mL (mean ± SD), of the 5 analytes for which the difference between WT- (white bars, n = 11) and SDS- (grey bars, n = 23) derived samples was statistically significant: GCS-F (**A**), IL-6 (**B**), IL-8 (**C**), IL-12 (**D**), MIP-1α (**E**). Analyses by Luminex^®^ xMap^®^ technology. * = p < 0.05; ** = p < 0.01

To investigate the possible implication of inflammation in SDS, we analyzed the expression of an enlarged panel of soluble mediators of inflammation in plasma obtained from peripheral blood of SDS patients and age-matched healthy donors, using Luminex^®^ xMap^®^ assays to simultaneously quantify 27 inflammatory markers (Fig. 2).

Results indicated that pro-inflammatory cytokines and chemokines, including G-CSF, IL-6, IL-8 and IL-12 and MIP-1α were significantly increased in SDS patients compared with healthy controls (Fig. 2). The increases observed in IL-6 and IL-8 are consistent with data in the literature (Bezzerri and Cipolli 2019; Bezzerri et al. 2016). In SDS patients, MIP-1α increased, though IL-6 remained within the physiological range.

Though levels of other cytokines and chemokines analyzed in SDS plasma tended generally to increase compared with healthy donors, these alterations were not statistically significant (Additional file 1: Figure S2).

**Fig. 3:**
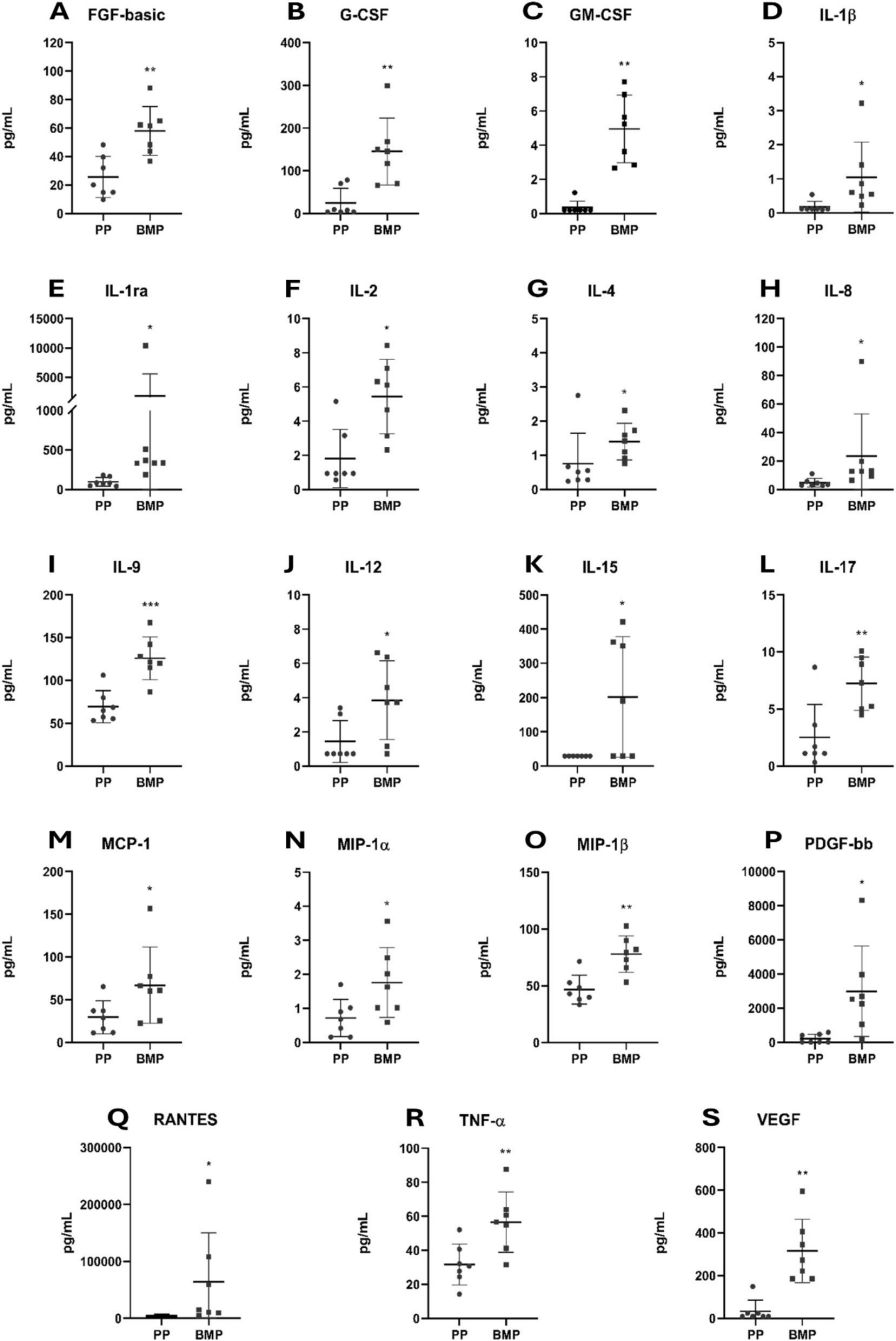
Expression profile of cytokines and chemokines in plasma obtained from peripheral and bone marrow blood of SDS patients. The scatter plots display the concentration, measured in pg/mL (mean ± SD), of 19 analytes whose levels differed significantly between peripheral blood plasma (PP, grey dots, n = 7) and bone marrow blood plasma (BMP, grey squares, n = 7) of SDS patients: FGF basic (**A**), G-CSF (**B**), GM-CSF (**C**), IL-1β (**D**), IL-1ra (**E**), IL-2 (**F**), IL-4 (**G**), IL-8 (**H**), IL-9 (**I**), IL-12 (**J**) IL-15 (**K**), IL-17 (**L**)**,** MCP-1 (**M**), MIP-1α (**N**), MIP-1β (**O**), PDGF-bb (**P**), RANTES (**Q**), TNF-α (**R**), VEGF (**S**). Analyses by Luminex^®^ xMap^®^ technology. * = p < 0.05; ** = p < 0.01; *** = p < 0.001

After confirming the increase in certain cytokines and chemokines in peripheral plasma of SDS patients compared to healthy donors, the secretome profile was analyzed in bone marrow plasma aspirates from SDS patients, given their characteristic bone marrow dysfunction (Fig. 3).

In fact, the bone marrow microenvironment, also known as the hematopoietic niche, is highly complex and tightly regulated by various factors such as the extracellular matrix, cell–cell interactions, physical parameters, and soluble factors (e.g., cytokines, chemokines, growth factors) (Yu and Scadden 2016).

The levels of cytokines and chemokines previously identified in blood obtained from SDS patients were statistically doubled in SDS bone marrow plasma (G-CSF, IL-8, MIP-1α) compared to peripheral blood obtained from the same patient, the only exception in this respect being IL-6 (Fig. 3).

In addition, further 16 cytokines and chemokines (FGF basic, GM-CSF, IL-1β, IL-1ra, IL-2, IL-4, IL-9, IL-12, IL-15, IL-17, MCP-1, MIP-1β, PDGF-bb, RANTES, TNF-α, VEGF) had statistically higher levels in SDS bone marrow plasma compared to SDS peripheral blood, while this trend was not identified in the 8 other cytokines and chemokines investigated (Additional file 1: Figure S3). This meant that cytokine and chemokine levels in the bone marrow did not match those in plasma, suggesting that bone marrow forms an independent compartment. Even at marrow stroma level, these inflammatory markers could favor ineffective hematopoiesis and progression to MDS and AML (Li and Calvi 2017).

**Fig. 4:**
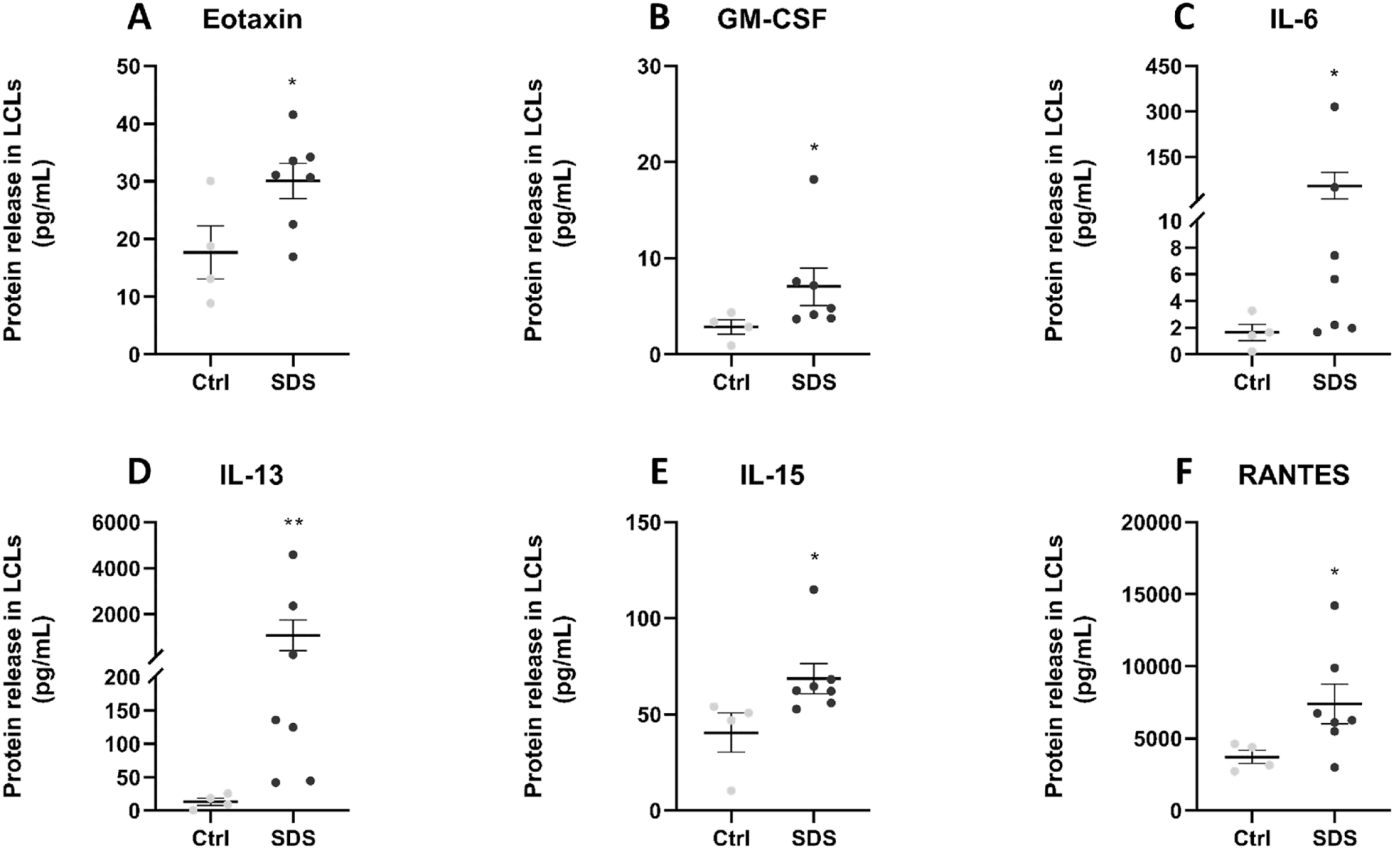
Release of cytokines and chemokines by LCLs isolated from healthy subjects and SDS patients. The scatter plots show the concentration, in pg/mL (mean ± SEM), of the 6 analytes for which the difference between Ctrl- (light grey dots, n = 4) and SDS- (grey dots, n = 7) derived samples was statistically significant: eotaxin (**A**), GM-CSF (**B**), IL-6 (**C**), IL-13 (**D**), IL-15 (**E**), RANTES (**F**). Analyses by Luminex^®^ xMap^®^ technology. * = p < 0.05; ** = p < 0.01

Similar trends were found in SDS LCLs (Fig. 4). All cytokines and chemokines that showed a higher level in SDS plasma (Fig. 2) were also increased in LCLs (Figs. 4 and Additional file 1: S4), reflecting their dysregulation. The statistically significant increase observed for IL-6, which is consistent with the literature (Vella et al. 2020), corroborates its action as soluble mediator with pleiotropic effect cells. In SDS LCLs, further soluble factors showing statistically significant increases were eotaxin, GM-CSF, IL-13, IL-15 and RANTES (Fig. 4).

### Everolimus effects on secretome profile in SDS LCLs

**Fig. 5:**
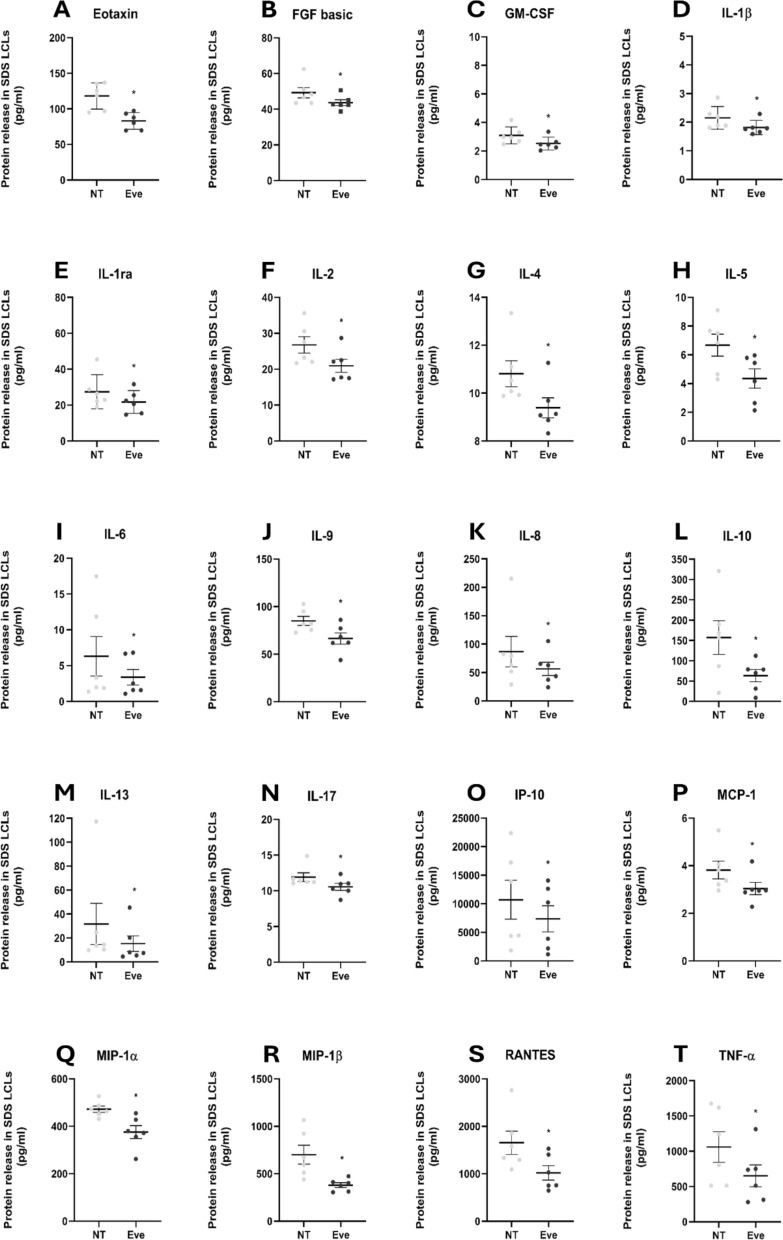
Effects of everolimus on the secretion of soluble inflammatory factors by LCLs. The scatter plots show the concentration, measured in pg/mL (mean ± SEM), of 20 analytes whose levels significantly differ in the supernatants of SDS-derived LCLs treated with everolimus 350 nM (Eve, grey dots, n = 6) or not treated (NT, light grey dots, n = 6): eotaxin (**A**), FGF basic (**B**), GM-CSF (**C**), IL-1β (**D**), IL-1ra (**E**), IL-2 (**F**), IL-4 (**G**), IL-5 (**H**), IL-6 (**I**), IL-8 (**J**), IL-9 (**K**) IL-10 (**L**), IL-13 (**M**)**,** IL-17 (**N**), IP-10 (**O**), MCP-1 (**P**)**,** MIP-1α (**Q**), MIP-1β (**R**), RANTES (**S**), TNF-α (**T**). Analyses by Luminex^®^ xMap^®^ technology. * = p < 0.05

Everolimus, a selective and clinically approved mTOR inhibitor, could prove useful as a means of ascertaining the mTOR pathway involvement in the cytokine and chemokine variations observed in SDS cells and plasma. The expression levels of eotaxin, FGF basic, GM-CSF, IL-1β, IL-1ra, IL-2, IL-4, IL-5, IL-6, IL-8, IL-9, IL-10, IL-13, IL-17, IP-10, MCP-1, MIP-1α, MIP-1β, RANTES and TNF-α (Fig. 5) were significantly reduced after everolimus treatment in SDS LCLs. Among these soluble inflammatory factors, four (eotaxin, GM-CSF, IL-6, IL-13) of six were up-regulated in everolimus untreated SDS LCLs (Fig. 4).

A general decrease was observed for all other factors in SDS LCLs treated with everolimus (Additional file 1: Figure S5), but without significant different levels.

A comparable trend was noted in experiments conducted on LCLs derived from healthy donors (HD), demonstrating no significant variation between untreated cells and those treated with everolimus (Additional file 1: Figure S6). In particular, for the untreated cells, IL-8 levels were lower in HD LCLs than SDS LCLs (Fig. 5), suggesting a reduced inflammatory activation; while IL-6 expression was generally similar between untreated HD and SDS LCLs, but a significant decrease was observed after everolimus treatment in SDS LCLs (Fig. 5 and Additional file 1: Figure S5). In general, everolimus treatment induced a significant reduction in cytokine secretion only in SDS LCLs, where mTOR pathway was hyperactivated and thus its inhibition resulted in a decrease of cytokine synthesis.

To assess the biological significance and implications of our findings, we conducted a functional enrichment analysis. The input consisted of the 20 genes coding for the proteins for which the Human Cytokine 27-plex Assay (Bio-Rad) showed a significant decrease in LCL supernatants after treatment with everolimus. The primary aim of this investigation was to decipher the functional implications of the identified proteins within the broader biological landscape. By submitting this gene list to the KEGG database and sorting the outcomes based on their FDR values in ascending order of significance, we found various enriched pathways. Of these, the “cytokine-cytokine receptor interaction pathway” is the most clearly enriched, with an FDR value of 3.8 × 10^–23^ and 15 genes involved (eotaxin, GM-CSF, IL-1β, IL-1ra, IL-2, IL-4, IL-5, IL-6, IL-8, IL-9, IL-10, IL-13, IL-17, IP-10, MCP-1). In second place, with 11 genes involved (eotaxin, GM-CSF, IL-1β, IL-4, IL-5, IL-6, IL-8, IL-13, IL-17, IP-10, MCP-1), is the “IL-17 signaling pathway” (FDR = 1.9 × 10^–20^), followed by pathways not directly linked with SDS but related to a chronic inflammatory condition (“inflammatory bowel disease” (Tavakoli et al. 2021) and “asthma” (Hammad and Lambrecht 2002)). Also worthy of mention is the “JAK-STAT signaling pathway”, in fifth place, with an FDR value of 1.1 × 10^–11^ and 8 genes involved (GM-CSF, IL-2, IL-4, IL-5, IL-6, IL-9, IL-10, IL-13). This finding shows that a substantial portion of these proteins exerts a profound influence on the pathway’s activation, thereby modulating cellular behaviors and survival. As delineated in the KEGG pathway map (Additional file 1: Figure S7), by binding their specific receptors, interleukins and growth factors trigger the activation of JAK proteins. Subsequently, JAKs induce the phosphorylation of STATs, followed by their dimerization and translocation to the nucleus, thereby modulating the expression of target genes.

Besides activating STATs, JAKs mediate the recruitment of other molecules such as the MAP kinases, PI3 kinase, AKT and mTOR, resulting in the activation of additional transcription factors. This intricate signaling cascade culminates in the activation of multiple genes involved in regulation of cell proliferation, apoptosis, cell cycle, cell survival, metabolism, and differentiation.

In addition, the enrichment analysis highlighted other pathways with pivotal roles in hematopoiesis, cancer, and cell differentiation. Taken together, these findings suggest that everolimus as a novel therapeutic solution, targeting mTOR, could reduce the levels of inflammation markers and interfere with additional pathways involved in the SDS pathophysiology.

### Differential miRNAS expression between SDS and normal LCLs

**Fig. 6:**
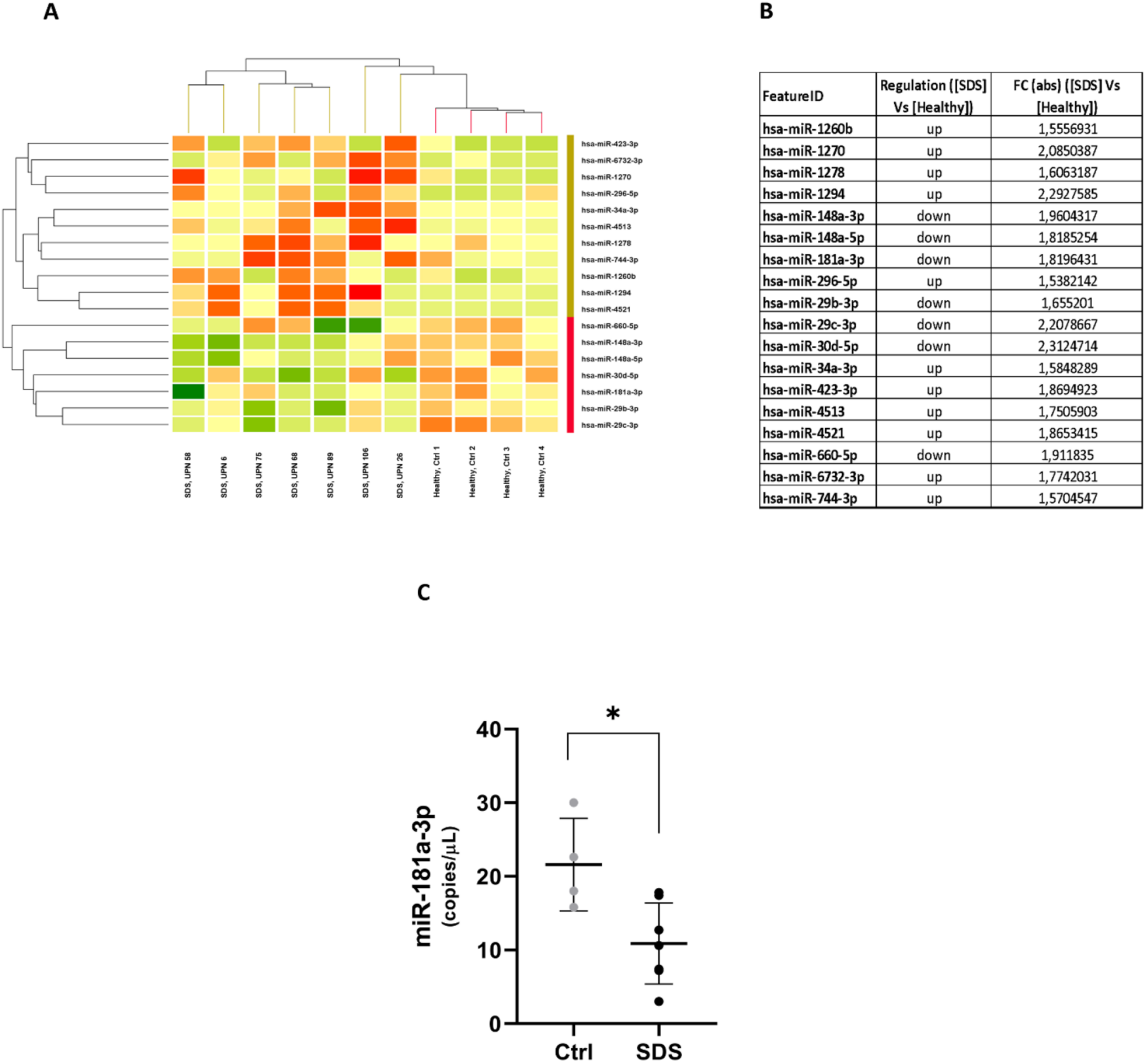
Differential expression levels of miRNAs obtained by NGS miRNome analysis and droplet digital RT-PCR validation. Heatmap (**A**) and expression fold-change levels (**B**) of microRNA in healthy lymphoblastoid cells (Ctrl) versus lymphoblastoid cells derived from SDS patients (SDS). Data showed microRNAs with fold change FC > 1.5. Green = down-regulation; red = up-regulation. (**C**) ddPCR, comparing control healthy cells and SDS cells in relation to the content of significantly dysregulated miRNA. * = p < 0.05

To identify miRNA (miR) signatures in SDS, we profiled the expression of seven SDS LCL and four healthy control LCL samples by microarray analysis, using the STAR algorithm and, as a reference genome, human miR sequences from the miRbase 22 (Fig. 6A and B). We observed 11 up-regulated miRs (hsa-miR-1260b, hsa-miR-1270, hsa-miR-1278, hsa-miR-1294, hsa-miR-296-5p, hsa-miR-34a-3p, hsa-miR-423-3p, hsa-miR-4513, hsa-miR-4521, hsa-miR-6732-3p, hsa-miR-744-3p) and 7 down-regulated miRs (hsa-miR-148a-3p, hsa-miR-148a-5p, hsa-miR-181a-3p, hsa-miR-29b-3p, hsa-miR-29c-3p, hsa-miR-30d-5p, hsa-miR-660-5p), in SDS cell cultures compared with controls (healthy donors) (Gasparello et al. 2019). The differential expressions of these miRs were then analyzed by ddPCR, showing a statistically significant dysregulation only for miR-181a-3p in SDS cells (Fig. 6C). This finding is consistent with the literature, where miR-181a-3p was reported as a diagnostic and prognostic biomarker for AML (Qiang et al. 2020) and hematologic malignancies, acting as a tumor suppressor in cellular division and differentiation (Roth and Cao 2015).

## Discussion

SDS is associated with hyperactivation of mTOR and STAT3 signaling pathways (Vella et al. 2020). In SDS, abnormal mTOR signaling may impact normal cellular metabolism, contributing to the clinical features of the syndrome. On the other hand, abnormalities in STAT3 signaling may affect immune responses, inflammation, and apoptosis. The dysregulation of mTOR-STAT3 signaling could contribute to the increased sensitivity to inflammation observed in individuals with SDS. The interplay between mTOR and STAT3 pathways has already been linked to inflammation (Furutani et al. 2020). It has been reported that SDS patients are more susceptible to inflammation than healthy individuals (Furutani et al. 2020). Besides activating STATs, JAKs mediate the recruitment of other molecules such as the MAP kinases, PI3 kinase, AKT, and mTOR, resulting in the activation of additional transcription factors that culminates in the expression of different genes involved in cell proliferation and differentiation. These signaling pathways are closely influenced by the inflammatory microenvironment (Furutani et al. 2020; Bezzerri et al. 2016), since many cytokines (e.g., IL-6, TNF-α, interferons) or growth factors may trigger their activation. Consistent with these findings, both in cell culture supernatants and in plasma samples we identified elevated levels of the pro-inflammatory soluble mediators IL-6, IL-8, IL-12, MIP-1α, G-CSF, which are closely correlated with mTOR/STAT3 pathways. Moreover, the higher levels of the analyzed cytokines, chemokines and growth factors in bone marrow plasma were probably due to the hematopoietic niche function and marked involvement of this district in SDS, the main clinical complications of which are bone marrow failure (BMF) and predisposition to myeloid malignancies (Furutani et al. 2022).

These results suggest that a constitutive chronic inflammation may play a role in the pathogenesis of SDS. Chronic inflammation is a major driver of carcinogenesis. Since 15–20% of SDS patients develop juvenile MDS or AML (Donadieu et al. 2012), our results provide an additional clue to the mechanisms underlying malignant transformation in SDS. We previously reported that rapamycin can suppress both mTOR and STAT3 in SDS, highlighting the cross-link between these two pathways (Bezzerri et al. 2016). Interestingly, here we show that the clinically approved analogue of rapamycin, everolimus (RAD001), is able to remarkably reduce inflammation in SDS cells, significantly inhibiting the release of 20 out of 27 pro-inflammatory soluble markers tested. These findings indicate that mTOR plays a pivotal role in SDS-related chronic inflammation.

As already reported, IL-6 can activate the mTOR and STAT3 pathways (Bezzerri et al. 2016), in turn generating an activation loop for IL-6, regulated by STAT3 transcription factor. IL-6 signaling is involved in several cellular processes, including inflammation, cell proliferation, and cancer (Lang 2005; Hansen 2020). In addition, IL-6 (also known as BSF2) can induce the maturation of B cells into memory B cells and plasma cells (Hirano et al. 1986). Importantly, elevated serum levels have been reported in patients with AML (Rosenberg et al. 2010), where it may stimulate blast cell proliferation and enhance G-CSF-dependent granulopoiesis. We previously reported elevated release of IL-6 in plasma of patients with SDS (Vella et al. 2020). Here, we extended the analysis to other pro-inflammatory mediators, including IL-8, TNF-α, MIP-1α and G-CSF, showing that they are also significantly up-regulated in SDS. Of note, it was recently reported that higher levels of IL-6 and TNFα signaling activity are associated with poorer outcomes in pediatric patients with AML (Bolouri et al. 2022). Our hypothesis is that these pro-inflammatory mediators are up-regulated in response to bone marrow failure, as a compensatory mechanism that forces bone marrow hematopoiesis. However, they may induce pro-leukemic signals that drive AML development. The constitutive chronic inflammation is a feature shared by several IBMFS, including Fanconi anemia (Dufour et al. 2003) and Diamond-Blackfan anemia (Kapralova et al. 2020). Although the inflammatory process has been associated with the senescence-associated secretory phenotype in Fanconi anemia (Helbling-Leclerc et al. 2021) ribosomopathies, and telomeropathies (Groarke et al. 2022), cellular senescence has been not yet fully characterized in SDS. However, mTOR hyper-activation and senescence-associated secretory phenotype (SASP)-related cytokines overexpression are in line with the hypothesis of a senescent phenotype in SDS as well. These aspects of SDS research are open to question, highlighting the need for further studies.

More importantly, we found a remarkable increase in the release of granulocyte colony-stimulating factor (G-CSF) in SDS plasma samples, possibly because of a compensatory process activated by SDS cells to overcome impaired myelopoiesis.

G-CSF and IL-6 are known to provide important survival and activation signals to monocytes/macrophages and neutrophil production, acting through STAT3 signaling intermediates (Gregory et al. 2010). Our results indicate that G-CSF plasma levels are significantly increased (by 50%) in SDS compared with healthy donors (Bezzerri et al. 2016; Vella et al. 2020). While elevated levels of IL-6 were already described in SDS (Vella et al. 2020), here we found an up-regulation of G-CSF release in SDS patients, which represents an important novelty. G-CSF is a major driver of myeloid lineage maturation in bone marrow, particularly in neutrophils. Patients with SDS are characterized by moderate to severe neutropenia, due to deficient myelopoiesis caused by excessive apoptosis of bone marrow hematopoietic progenitors (Dror et al. 2001) and increased p53 signaling (Elghetany and Alter 2002). This immunodeficiency is associated with high susceptibility to bacterial infections, particularly in the early stages of life. It follows that the clinical use of recombinant G-CSF (i.e., filgrastim) has been considered as a useful therapeutic option for SDS patients. However, the chronic administration of G-CSF is detrimental, because of its leukemogenic adverse effects (Rosenberg et al. 2006, 2010). Since elevated levels of IL-6 are associated with leukemic transformation and poor prognosis both in animal models of MDS and in patients with AML (Mei et al. 2022; Sanchez-Correa et al. 2013), respectively, the increased levels of both IL-6 and G-CSF provide a crucial clue on the mechanisms underlying development of MDS and leukemic transformation in SDS patients.

IL-8 is a pro-inflammatory cytokine mainly released by monocytes, endothelial cells, and a wide range of epithelial cells. It regulates the bone marrow microenvironment and promotes clonal expansion (Baggiolini and Clark-Lewis 1992). Generally, IL-8 is involved in infection response and acts as a driver of inflammation and carcinogenesis due to its pleiotropic functions. IL-8 is involved in angiogenesis and stimulates cancer cell proliferation (Bakouny and Choueiri 2020). More importantly, higher plasma levels of IL-8 are associated with poorer outcomes in patients with various types of cancer (Alfaro et al. 2017 Nov). In this respect, we found that IL-8 plasma levels are significantly up-regulated in peripheral blood of SDS patients, thus strengthening the hypothesis that the particular pro-inflammatory environment is dominated by pro-tumoral mediators. Elevated IL-8 levels were found in patients with AML, impacting niche formation and correlating with poor prognosis (Kuett et al. 2015). It follows that the increased release of IL-8 observed in SDS may be an additional factor promoting leukemogenesis.

Most importantly, we observed that plasma levels of IL-1β, IL-8, IL-9, TNF-α, GM-CSF, MIP-1α, and MCP-1 are even increased in the bone marrow compartment, compared with peripheral blood. These data suggest that BM is the main source of pro-inflammatory soluble mediators. In addition, the higher expression of growth factors in BM, such as GM-CSF, might reflect their local regulatory role in the hematopoiesis process. However, their constitutive up-regulation raises an important red flag in relation to the latent leukemogenic process.

In the last two decades, a plethora of miRNAs have been linked to different cellular processes such as apoptosis and cell proliferation (Kawashima et al. 2023b). Furthermore, miRNAs have also been described as regulators of leukemogenesis (Stavast et al. 2018). Interestingly, miR-181a-3p overexpression has been associated with STAT3 dysregulation, increased ERK1/2 activation and resistance to Fas-mediated apoptosis (Assmann et al. 2022). Here, we described down-regulation of miR-181a-3p in SDS patients, suggesting its involvement in regulation of the pro-inflammatory and leukemogenic cellular programs downstream from lost *SBDS* expression. SDS is characterized by a Fas-mediated increase in apoptosis of bone marrow myeloid progenitor cells (Dror et al. 2001). The reduction of miR-181a-3p may therefore provide the missing link between absence of SBDS expression and excessive apoptosis of bone marrow hematopoietic progenitors. Moreover, AML patients with higher miR-181a-3p expression at diagnosis had a better prognosis than those with lower levels (Qiang et al. 2020; Roth and Cao 2015; Schwind et al. 2010). On the other hand, miRNA-181a-3p expression was significantly decreased in AML patients who achieved a complete response after induction chemotherapy (Qiang et al. 2020). The low miR-181a-3p expression in our SDS patients could correlate with potential AML development (Weng et al. 2015). This suggests that miR-181a-3p may play a role in the SDS pathogenesis, potentially contributing to the common cytokine signature observed in IBMFS (Kawashima et al. 2023b), such as SDS, in response to bone marrow failure. The common cytokine signature may be a result of the body's attempt to compensate for the lack of blood cell production.

## Conclusion

While the precise involvement of the analyzed cytokines and chemokines in SDS has not yet been investigated, these preliminary results suggest that some of these inflammatory factors (such as GCS-F and IL-8) could be involved in the molecular pathophysiology of SDS and, therefore, be classified as molecular targets for development of a specific therapeutic strategy. Furthermore, this study underlines the involvement of mTOR-mediated constitutive systemic inflammation in SDS, suggesting that the main source of pro-inflammatory mediators is localized in the bone marrow. This chronic inflammation may be a major driver of leukemogenesis in SDS. Ultimately, this work paves the way for extensive investigation of inflammatory markers in other IBMFS characterized by cancer predisposition, potentially providing novel diagnostic and prognostic targets.

## Supplementary Information

Additional file 1.

## Data Availability

No datasets were generated or analysed during the current study.

## Abbreviations

SDS: Shwachman-Diamond Syndrome
MDS: Myelodysplastic syndrome
AML: Acute myeloid leukemia
mTOR: Mammalian target of rapamycin
STAT-3: Signal transducer and activator of transcription
LCLs: Lymphoblastoid cells
IBMFS: Bone marrow failure syndrome
SBDS: Shwachman-Bodian-diamond syndrome
CRMO: Chronic recurrent multifocal osteomyelitis
RT-PCR: Real time-PCR
ddPCR: Droplet digital PCR
NGS: Next generation sequencing
miRNAs: MicroRNAs
mRNA: Messenger RNA
RISC: RNA-induced silencing complex
FDR: False discovery rate
KEGG: Kyoto encyclopedia of genes and genomes
AutoDG: Automated droplet generator
IBMFS: Inherited bone marrow failure syndromes
SASP: Senescence-associated secretory phenotype
