# Supplementary material for: Constitutive systemic inflammation in Shwachman-Diamond Syndrome
Source: Mol Med. 2025 Feb 28;31:81. doi: 10.1186/s10020-025-01133-5 (PMC11869671; doi:10.1186/s10020-025-01133-5)
Supplement: Supplementary file 1 — Additional file 1. [file 10020_2025_1133_MOESM1_ESM.docx]

**
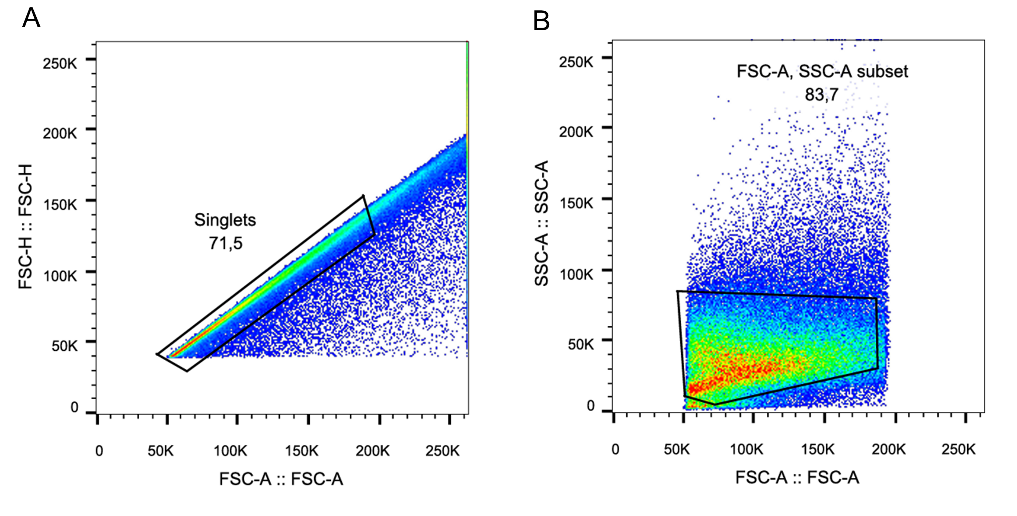
**

**Figure S1.** **Optimization of flow cytometry analysis in fixed and permeabilized LCL as performed in Figure 1A. A**, doublet discrimination. Doublets (blue) were isolated from single cells (green) by plotting FSC height vs FCS area. **B**, gating strategy based on morphological analysis.

**
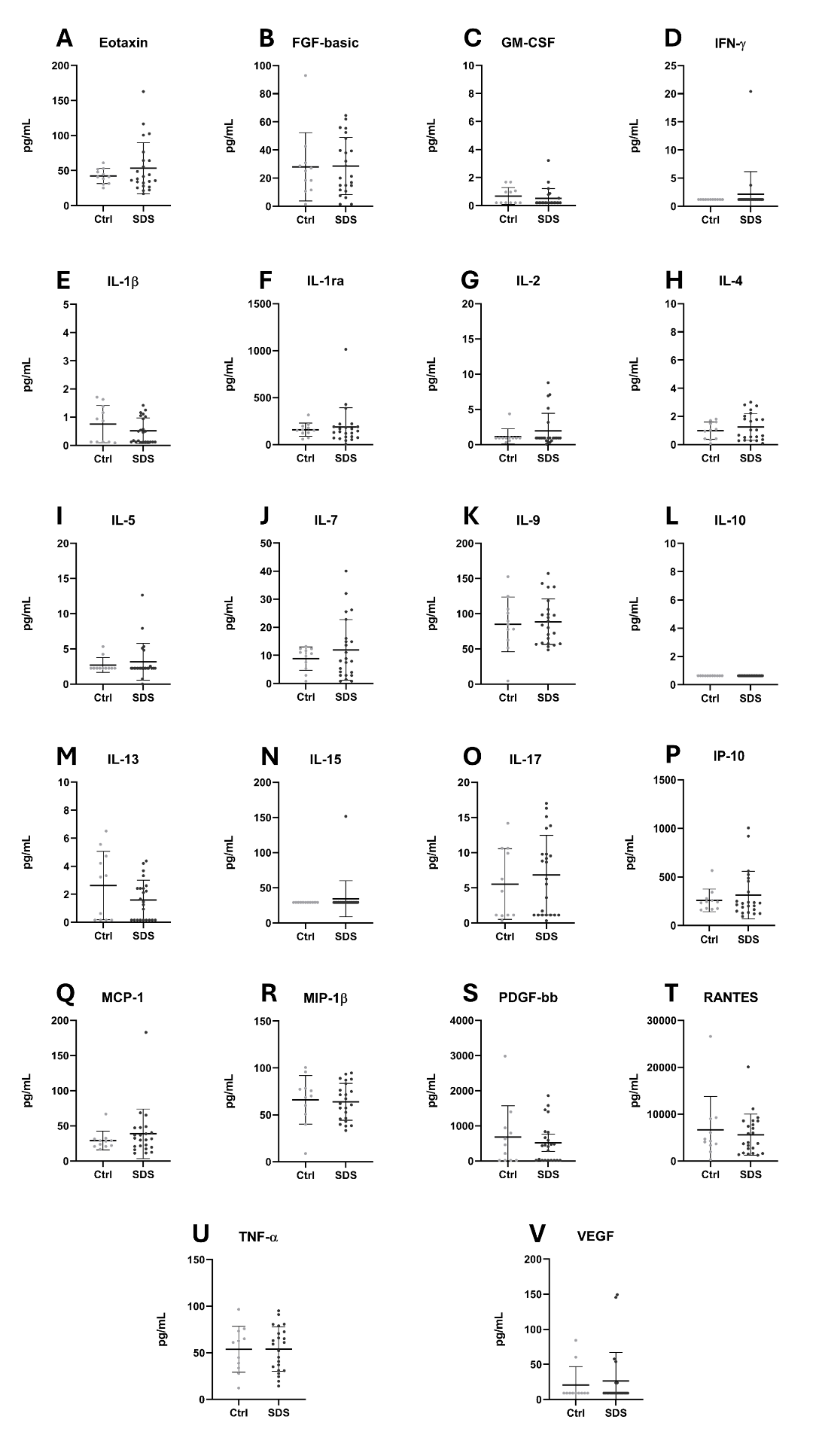
**

**Figure S2. Expression profile of cytokines and chemokines in plasma samples obtained from peripheral blood of healthy subjects and SDS patients.** The scatter plots show the concentration, in pg/mL (mean ± SD), of the 22 analytes for which the difference between WT (light grey dots, n=11) and SDS (grey dots, n=23) derived samples was not statistically significant: Eotaxin (A), FGF basic (B), GM-CSF (C), IFN-γ (D), IL-1β (E), IL-1ra (F), IL-2 (G), IL-4 (H), IL-5 (I), IL-7 (J) IL-9 (K) IL-10 (L), IL-13 (M), IL-15 (N), IL-17 (O) IP-10 (P), MCP-1 (Q), MIP-1β (R), PDGF-bb (S), RANTES (T), TNF-α (U), VEGF (V). Analysis were conducted using Luminex® xMap® technology.


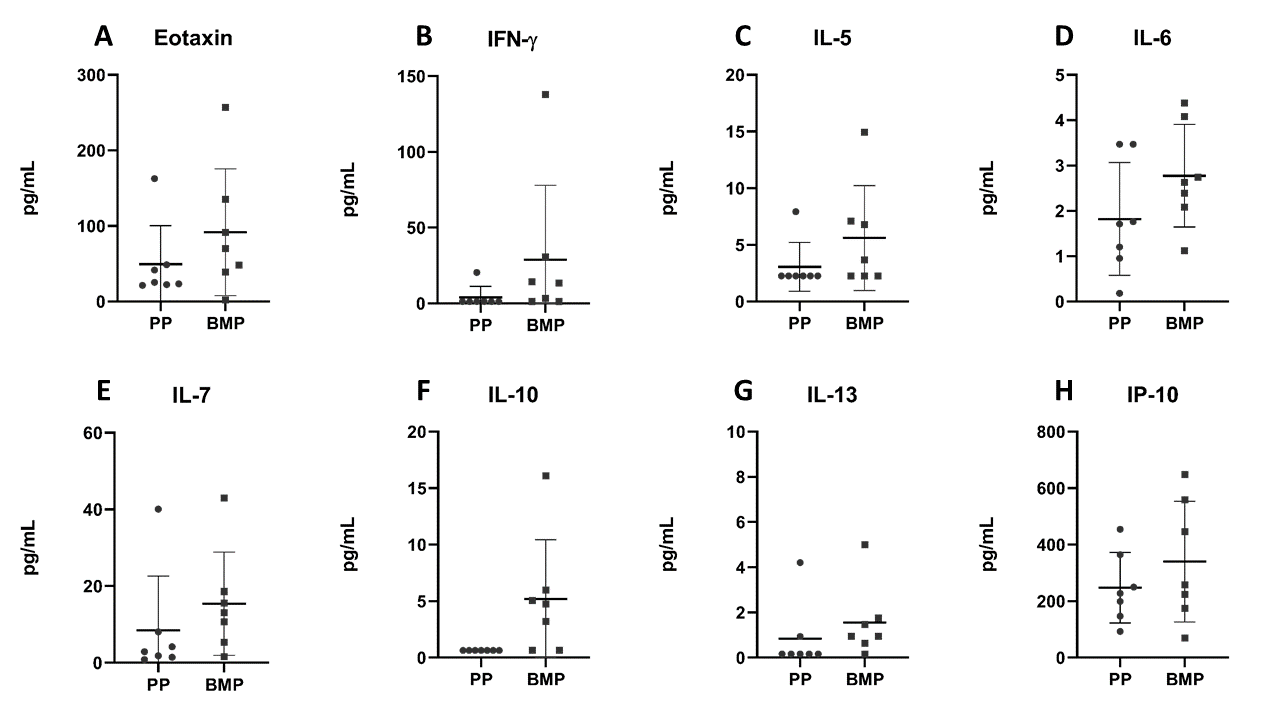


**Figure S3. Expression profile of cytokines and chemokines in plasmas obtained from peripheral and bone marrow blood of SDS patients.** The scatter plots show the concentration, measured in pg/mL (mean ± SD), of 8 analytes whose levels do not differ significantly between peripheral blood plasma (PP, grey dots, n=7) and the bone marrow blood plasma (BMP, grey squares, n=7) of SDS patients: Eotaxin (**A**), IFN-γ (**B**), IL-5 (**C**), IL-6 (**D**), IL-7 (**E**), IL-10 (**F**); IL-13 (**G**), IP-10 (**H**). Analysis were conducted using Luminex® xMap® technology.


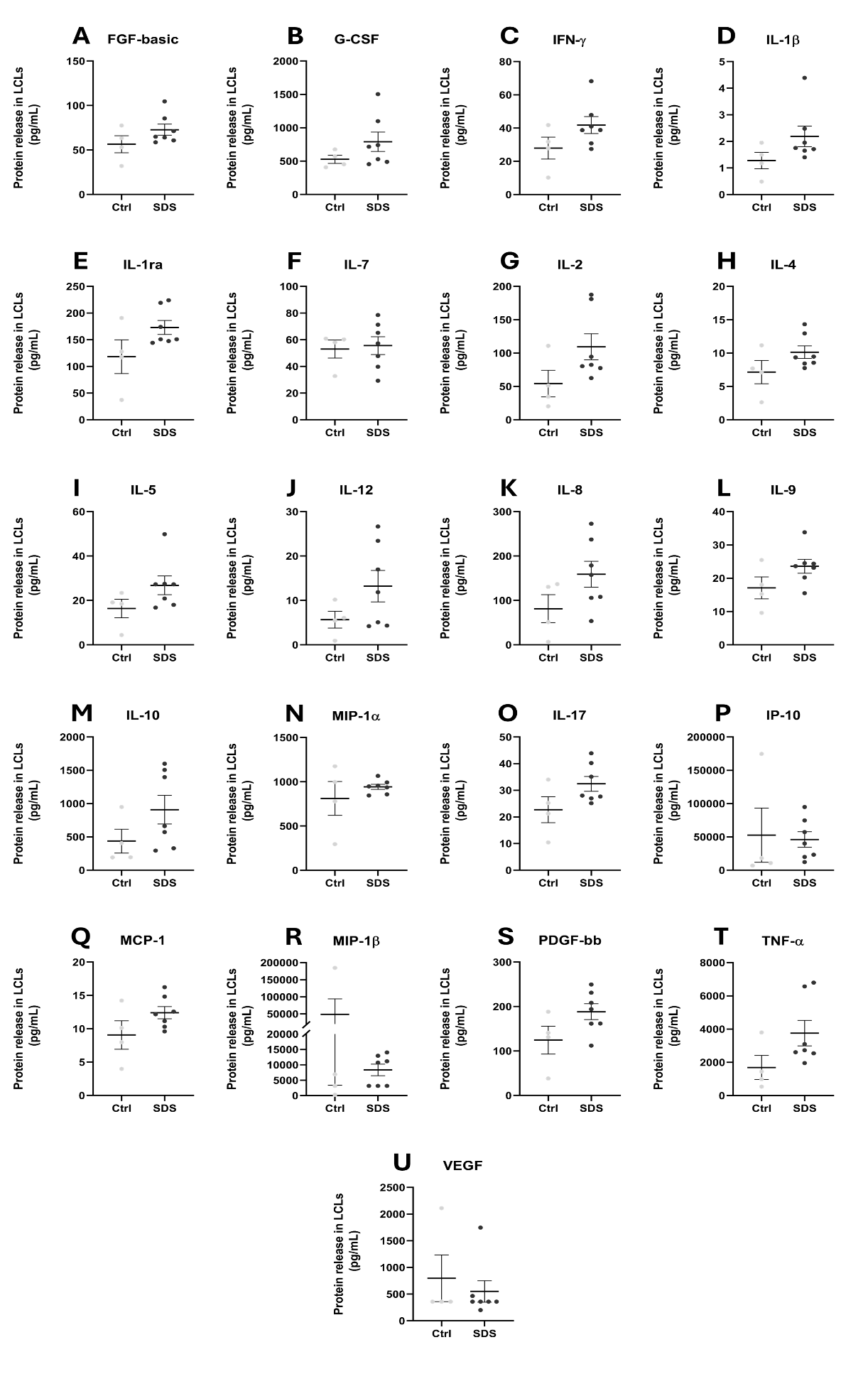


**Figure S4.** **Release of cytokines and chemokines by LCLs isolated from healthy subjects and SDS patients.** The scatter plots show the concentration, in pg/mL (mean ± SEM), of the 21 analytes for which the difference between Ctrl (light grey dots, n=4) and SDS (grey dots, n=7) derived samples was not statistically significant: FGF-basic (**A**), G-CSF (**B**), IFN-γ (**C**), IL-1β (**D**), IL-1ra (**E**), IL-2 (**F**), IL-4 (**G**), IL-5 (**H**), IL-7 (**I**), IL-8 (**J**), IL-9 (**K**), IL-10 (**L**), IL-12 (**M**), IL-17 (**N**), IP-10 (**O**), MCP-1 (**P**), MIP-1α (**Q**), MIP-1β (**R**), PDGF-bb (**S**), TNF-α (**T**), VEGF (**U**). Analysis were conducted using Luminex® xMap® technology.


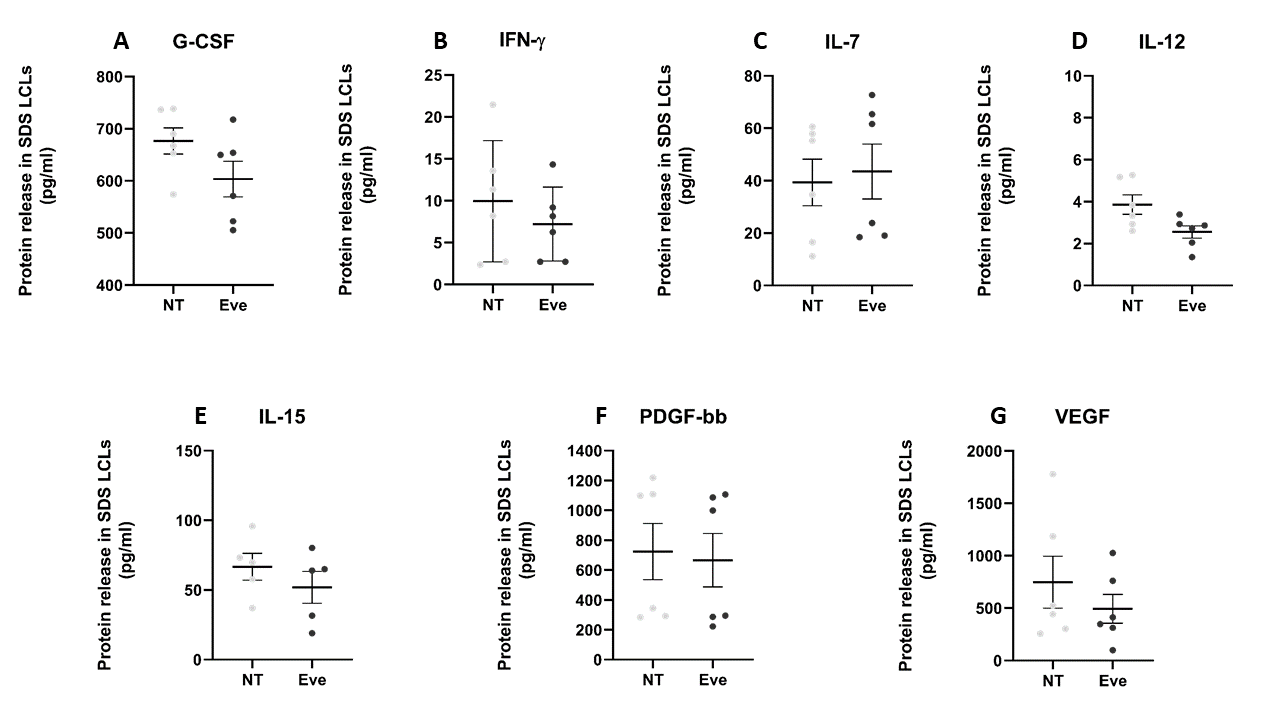


**Figure S5. Effects of Everolimus on the secretion of soluble inflammatory factors by LCLs.** The scatter plots show the concentration, measured in pg/mL (mean ± SEM), of 7 analytes whose levels do not differ significantly in the supernatants of SDS derived LCLs treated with Everolimus 350nM (Eve, grey dots, n=4) or not treated (NT, light grey dots, n=4): G-CSF (**A**), IFN-γ (**B**), IL-7 (**C**), IL-12 (**D**), IL-15 (**E**). PDGF-bb (**F**), VEGF (**G**). Analysis were conducted using Luminex® xMap® technology.


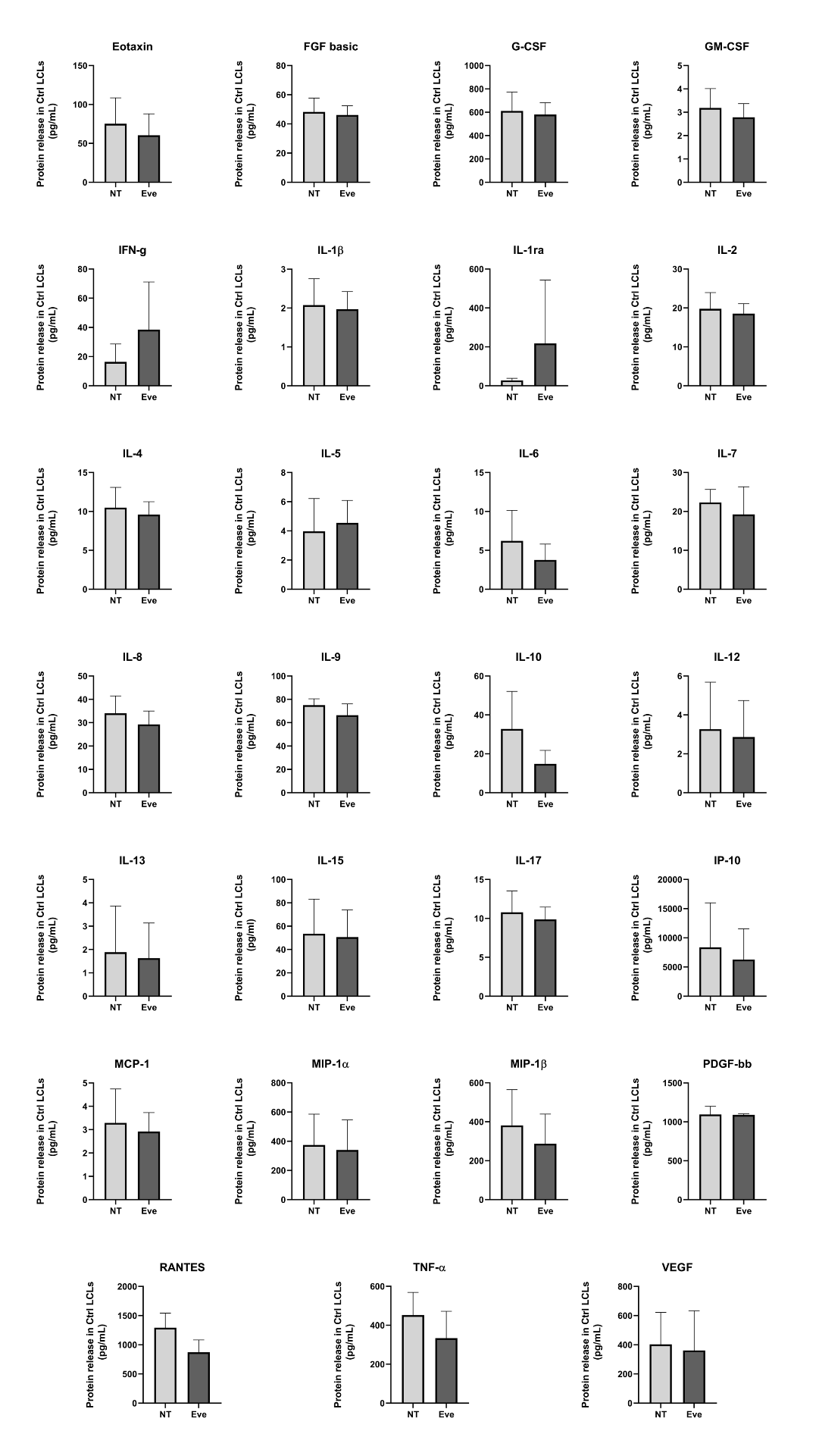
**Figure S6. Effects of everolimus on the secretion of soluble inflammatory factors by healthy donors (HD) LCLs.** The histograms show the concentration, measured in pg/mL (mean ± SEM), of 27 analytes detected in the supernatants of healthy donor (Ctrl) derived LCLs treated with Everolimus 350nM (Eve, grey bars, n=3) or not treated (NT, light grey bars, n=3): Eotaxin (**A**), FGF basic (**B**), GM-CSF (**C**), IL-1β (**D**), IL-1ra (**E**), IL-2 (**F**), IL-4 (**G**), IL-5 (**H**), IL-6 (**I**), IL-8 (**J**), IL-9 (**K**) IL-10 (**L**), IL-13 (**M**), IL-17 (**N**), IP-10 (**O**), MCP-1 (**P**), MIP-1α (**Q**), MIP-1β (**R**), RANTES (**S**), TNF-α (**T**). Analysis were conducted using Luminex® xMap® technology.


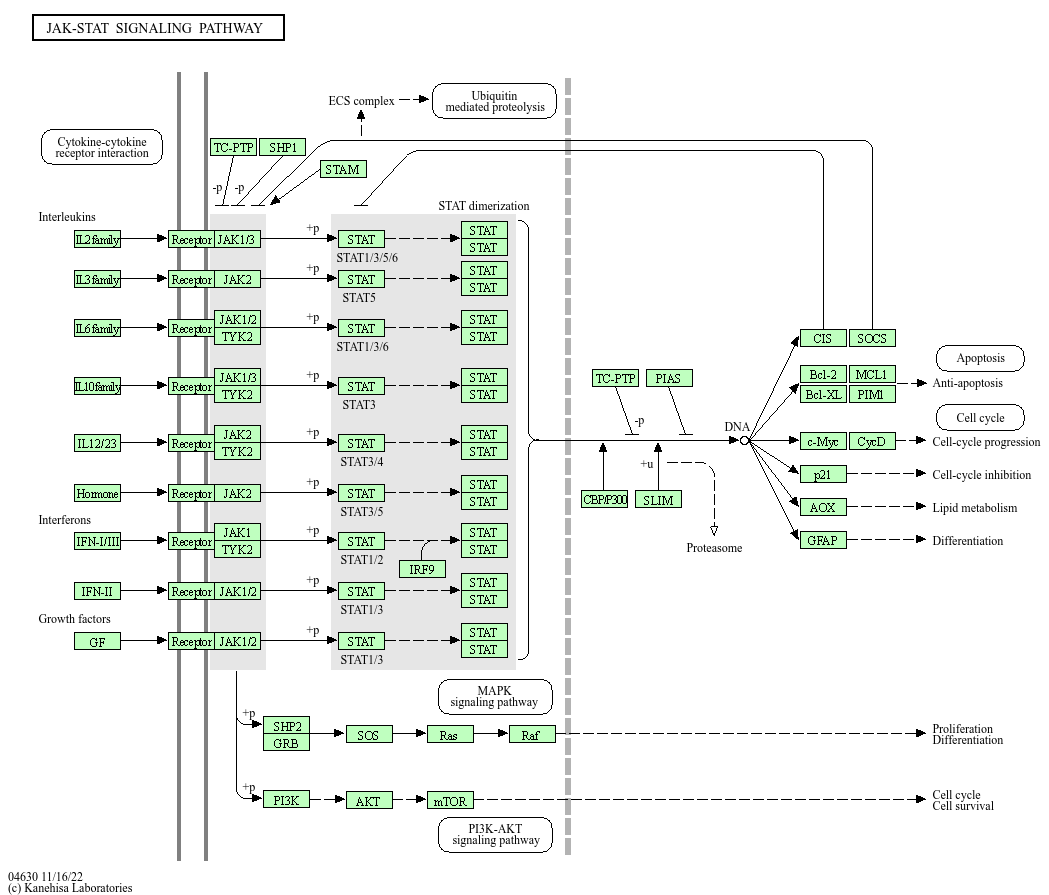


**Figure S7.**  **Kegg pathway map of JAK-STAT signaling pathway.** The JAK-STAT signaling pathway resulted among the most represented in the functional enrichment analysis (FDR = 1.3x10^-12^), preceded only by the cytokine-cytokine receptor interaction pathway (FDR = 5.7x10^-13^). As shown in the KEGG pathway map these stimuli, binding their specific receptor, induce a series of signal cascades which lead to influence both cell proliferation and apoptosis, involving also MAPK and PI3K-AKT signaling pathways.
